# Supplementary figures and images for: Hormonal Regulation and Transcriptomic Insights into Flower Development in Hydrangea paniculata ‘Vanilla Strawberry’
Source: Plants (Basel). 2024 Feb 8;13(4):486. doi: 10.3390/plants13040486 (PMC10893276; doi:10.3390/plants13040486)

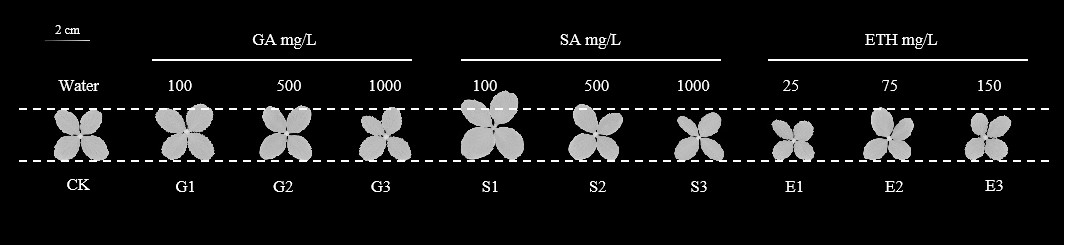

Supplement: Supplementary file 1 [file plants-13-00486-s001.zip › Figure S1.tif]
